# Ubiquitin-specific protease 7 regulates myocardial ischemia/reperfusion injury by stabilizing Keap1

**DOI:** 10.1038/s41420-022-01086-2

**Published:** 2022-06-16

**Authors:** Qiong Xu, Mingke Liu, Jielei Gu, Sisi Ling, Xiaolin Liu, Zhenyu Luo, Yangshuo Jin, Renjie Chai, Wenchao Ou, Shiming Liu, Ningning Liu

**Affiliations:** Department of Cardiology, Guangzhou Institute of Cardiovascular Disease, Guangdong Key Laboratory of Vascular Diseases, State Key Laboratory of Respiratory Disease, the Second Affiliated Hospital, Guangzhou Medical University, Guangzhou, 510260 China

**Keywords:** Myocardial infarction, Translational research

## Abstract

Myocardial ischemia/reperfusion (I/R) injury is a complex pathological process that is still not fully understood. The oxidative stress response has a critical role in the occurrence and progression of myocardial ischemia/reperfusion injury. This study investigated the specific mechanism of ubiquitin-specific protease 7 (USP7) regulation of myocardial ischemia/reperfusion injury from the perspective of proteasome degradation and its relation with the Keap1 pathway, a vital regulator of cytoprotective responses to endogenous and exogenous stress induced by reactive oxygen species (ROS) and electrophiles. Our data indicated that USP7 expression is increased during myocardial ischemia/reperfusion injury in mice, while its inhibiting suppressed the generation of oxygen free radicals and myocardial cell apoptosis, reduced myocardial tissue damage, and improved heart function. Mechanistically, USP7 stabilizes Keap1 by regulating its ubiquitination. Taken together, these findings demonstrate the potential therapeutic effect of USP7 on myocardial ischemia/reperfusion injury.

## Introduction

Myocardial ischemia/reperfusion (I/R) injury was first proposed in 1960 by Jennings et al. who examined the canine heart as a coronary ligation model [1, 2]. Myocardial reperfusion injury has a significant effect on cellular function, including oxidative stress responses that generate large amounts of oxygen free radicals [3, 4], lead to intracellular calcium overload [5], affect mitochondrial permeability transition pore (MTPT) opening [6, 7], and cause pH changes. During reperfusion, Harmful chemicals are produced and accumulated in the myocardium, thus causing damage to the myocardium [8–11]. Oxidative stress occurs when reactive oxygen species (ROS) levels exceed the antioxidant defense system’s capacity. When the production of ROS exceeds the body’s ability to remove hydrogen peroxide, the balance between oxidation and the antioxidant system is disrupted, leading to cell damage and death [12–14]. In addition, the production of ROS by the xanthine oxidase system, which causes oxidative stress, is strongly linked to cardiomyocyte injury and greater myocardial injury following acute myocardial infarction [15–18]. Preclinical studies suggested that lethal reperfusion injury accounts for up to 50% of the final size of myocardial infarction [19].

Keap1/Nrf2 pathway is the vital regulator of cytoprotective responses to endogenous and exogenous stress induced by ROS and electrophiles. Meanwhile, it is also a key signaling pathway for reducing the size of the infarction and protecting cardiac function after I/R [20, 21]. Keap1 can suppress the Nrf2 pathway. In physiological conditions, two Keap1 molecules bind to Nrf2, and Keap1 retains Nrf2 in the cytoplasm, blocking Nrf2 and shortening its half-life by targeting it for proteasome degradation. However, during oxidative stress, Keap1 is structurally altered, so Nrf2 cannot be inhibited and is further translocated into the nucleus [21, 22].

Deubiquitinating enzymes (DUBs) are a component of the ubiquitin-proteasome system (UPS), which can perform cell biology functions by cutting the ubiquitin chain of the substrate [23, 24]. Studies have shown that DUBs have a role in cardiovascular events, such as myocardial hypertrophy, atherosclerosis, myocardial fibrosis, myocardial infarction, and myocardial ischemia/reperfusion injury, among others [25–29]. The USP family includes the ubiquitin-specific protease 7 (USP7), known as the herpesvirus-associated ubiquitin-specific protease (HAUSP). It is mainly located in the nucleus, where it controls transcription, DNA damage response, epigenetic regulation of gene expression, viral infection, and immunological response, among other cellular functions. In addition, abnormal expression and activity of USP7 have been associated with biological activities such as apoptosis, inflammation, and life cycle regulation [30–32]. Xue et al. conducted cell in vitro experiments and found that USP7 significantly increases under hypoxia in cardiomyocytes, while its inhibition can reduce the myocardial damage caused by continuous hypoxia, which indicates that USP7 is effective in myocardial damage [33]. However, so far, only a few studies reported on the involvement of USP7 in myocardial ischemia/reperfusion injury and its molecular mechanism. In order to provide an innovative theoretical basis for the prevention and treatment of myocardial ischemia/reperfusion injury, this study investigated the specific mechanism of USP7 regulation of myocardial ischemia/reperfusion injury from the perspective of proteasome degradation and its relation with the Keap1 pathway.

## Results

### The expression of USP7 increases in myocardial infarction tissue

**Fig. 1:**
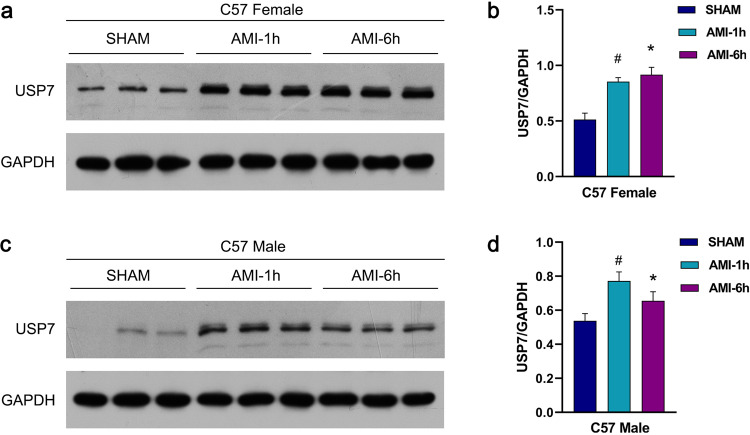
Expression of USP7 in myocardial infarction. **a**, **b** The expression of USP7 in female mice after AMI detected using a Western blot. **c**, **d** The expression of USP7 in male mice after AMI detected using a Western blot. ^#^*P* < 0.05, mean standard deviation (*n* = 3), compared to the SHAM group. *P < 0.05, mean standard deviation (*n* = 3), compared to the SHAM group.

In this study, the left anterior descending coronary artery (LAD) was ligated in C57BL/6 J mice to establish an acute myocardial infarction model. The expression of USP7 in heart tissue was detected by Western blot. As shown in Fig. 1a, b, USP7 protein expression was upregulated in male mice 1 h and 6 h after myocardial infarction. In addition, considering the estrogen’s cardioprotective effect [34], we performed the same surgery in female mice. As a result, USP7 expression was increased in female mice (Fig. 1c, d), which suggests that USP7 is a regulator of myocardial ischemia.

### The expression of USP7 increases in myocardial ischemia/reperfusion injury

**Fig. 2:**
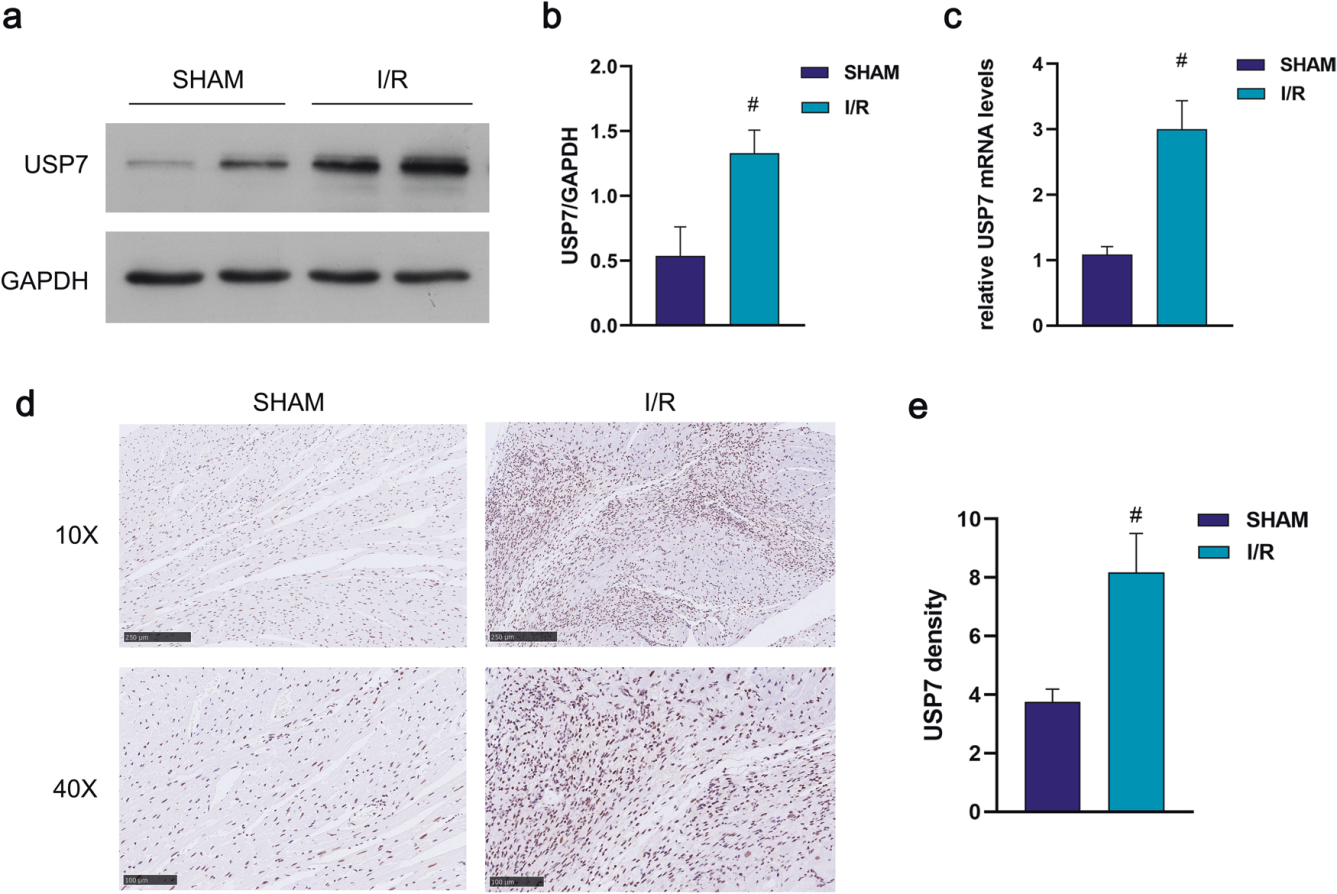
Expression of USP7 in myocardial ischemia/reperfusion. **a**, **b** The cardiac tissue was collected after 30 min of ischemia and 2 h of reperfusion, then the protein expression of USP7 was detected by Western blot. **c**, **d** Immunohistochemistry. **e** RT-qPCR. ^#^*P* < 0.05, mea ± standard deviation (*n* ≥ 3), compared with the SHAM group.

With reference to previous studies [33], we preliminarily identified USP7 as a regulatory factor in myocardial ischemia. To further investigate whether USP7 is involved in myocardial ischemia/reperfusion injury, we established a mouse I/R model and detected USP7 expression using RT-QPCR, immunohistochemical staining, and Western blot. As shown in Fig. 2, a substantial increase in USP7 expression was found in the I/R group compared to the SHAM group. These findings imply that USP7 may partcipate in myocardial ischemia/reperfusion injury regulation.

### Inhibition of USP7 reduces the apoptosis of cardiomyocytes

**Fig. 3:**
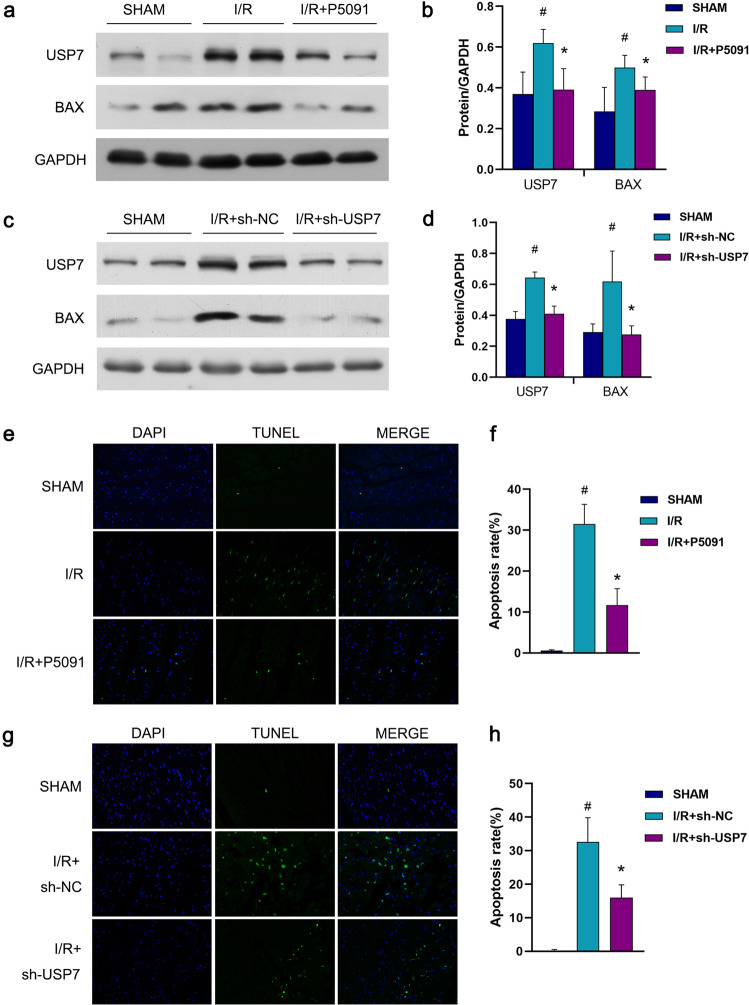
Cardiomyocyte apoptosis can be reduced by inhibiting USP7. **a**, **b** sh-USP7 was injected into the myocardium of mice, and the myocardial ischemia/reperfusion model was established seven days later. Then the myocardial tissue was removed, and the Western blot assay was used to determine the protein expression of USP7 and BAX. **c**, **d** P5091 was intraperitoneally injected into mice for three consecutive days, and then the myocardial ischemia/reperfusion model was established. The western blot assay detected the protein expression of USP7 and BAX in mouse myocardium. **e**, **f** Mice were given the same treatment as in (**a**, **b**), representative TUNEL staining images indicating the apoptotic rate, scar bar = 50 μm. **g**, **h** Mice were given the same treatment as in (**c**, **d**), representative TUNEL staining images indicating the apoptotic rate, scar bar = 50 μm. ^#^*P* < 0.05, mean ± standard deviation (*n* = 3), compared with the SHAM group. *P < 0.05, mean ± standard deviation (*n* = 3), compared with the I/R group.

One of the major mechanisms causing myocardial ischemia/reperfusion injury is apoptosis. The *BAX* gene (Bcl-2 Associated X-protein) is a member of the *Bcl-2* gene family that promotes apoptosis. We employed a mouse I/R model and used sh-RNA and USP7 inhibitor P5091 to inhibit USP7 and a Western blot to measure the expression of pro-apoptotic protein BAX and examined whether USP7 can regulate myocardial ischemia/reperfusion damage by altering apoptosis. Meanwhile, TUNEL staining was employed to evaluate cardiomyocyte apoptosis. The results show that the protein expression levels of USP7 and BAX in the I/R group were higher than in the SHAM group, while they were reduced when USP7 was inhibited (Fig. 3a–d). Similarly, in the I/R group, TUNEL staining results showed a substantial increase in the number of positive cells, while inhibition of USP7 reduced apoptosis (Fig. 3e–h). These results suggest that inhibition of USP7 can suppress apoptosis of cardiomyocytes.

### Inhibition of USP7 improves cardiac function

**Fig. 4:**
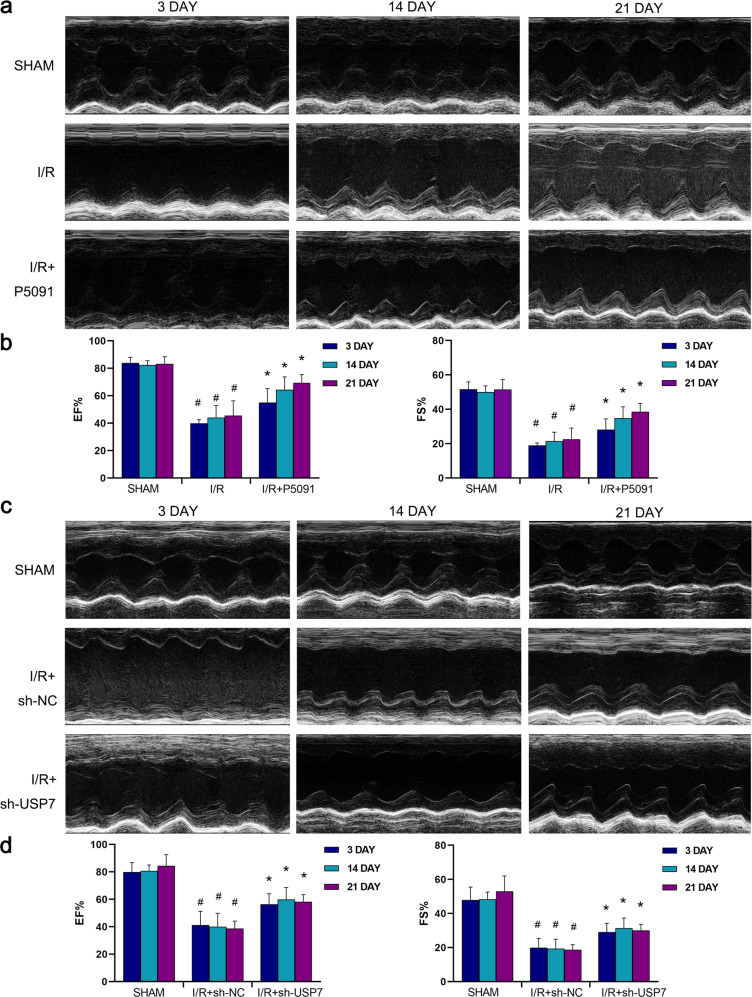
Mice with cardiac dysfunction were treated by inhibiting USP7. **a**, **b** P5091 was intraperitoneally injected into mice for three consecutive days, and ultrasound Doppler was used to detect the EF% (ejection fraction) and FS% (fraction shortening) 3, 4, and 21 days after the operation. **c**, **d** sh-USP7 was injected into the myocardium of mice, and ultrasound Doppler was used to detect the EF and FS 3, 4, and 21 days after the operation. ^#^Compared with the SHAM group, *Compared with the I/R group, *P* < 0.05, mean ± standard deviation (*n* = 3).

To investigate whether inhibition of USP7 could improve impaired cardiac function in mice, we performed echocardiography on the mice. As shown in Fig. 4a, b, mice in the I/R group had different degrees of cardiac dysfunction, with lower ejection fraction (EF%) and shortening fraction (FS%) than normal values. We dynamically observed the cardiac function of the mice after treatment with P5091 for I/R and discovered that the EF% and EF% of the heart were improved on day 3, day 14, and day 21. Mice treated with sh-USP7 showed the same results (Fig. 4c, d). This suggests that inhibition of USP7 can improve cardiac function in mice in a lasting and effective way.

### Inhibition of USP7 reduces myocardial injury

**Fig. 5:**
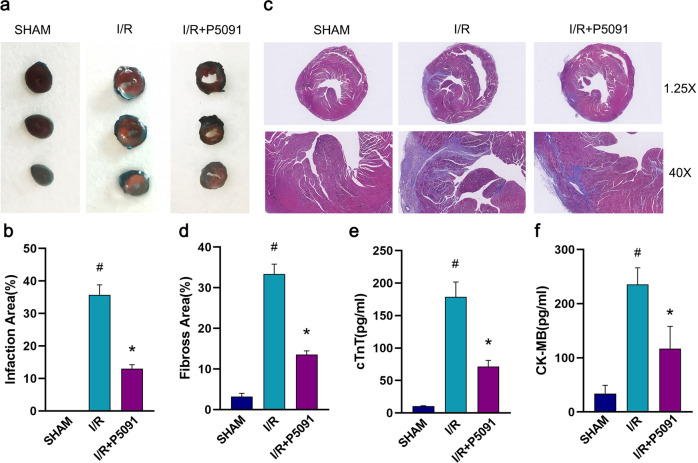
Mice myocardial injury was reduced when USP7 was inhibited. **a**, **b** P5091 was intraperitoneally injected into mice for three consecutive days, then after 24 h of reperfusion, Evans blue/TTC staining was used to stain the hearts of mice. Representative images are presented. **c**, **d** Masson staining was used to detect myocardial fibrosis in mice. Myocardial collagen fibers are shown in blue and myocardial fibers are colure in red. Representative images are shown. **e**, **f** The expression levels of CK-MB and cTnT in serum of mice. ^#^Compared with the SHAM group, *Compared with the I/R group, *P* < 0.05, mean ± standard deviation (*n* = 3).

To determine whether USP7 inhibition can alleviate organic damage, myocardial infarction area and myocardial fibrosis were measured by Evans Blue/TTC staining and Masson’s trichrome staining, respectively. The results showed significant myocardial infarction in mice during myocardial ischemia/reperfusion, and the final myocardial infarct size was reduced after inhibition of USP7 (Fig. 5a, b). In addition, myocardial fibers in the I/R group were disordered, and collagen deposition was abundant, whereas inhibition of USP7 reduced the effect of I/R on myocardial fibers (Fig. 5c, d). Furthermore, blood levels of creatine kinase-MB (CK-MB) and cardiac Troponin T (cTnT), both indicators of myocardial injury, were considerably higher in the I/R group but much lower following USP7 suppression.

### Inhibition of USP7 reduces ROS production through Nrf2/Keap1 signaling pathway

**Fig. 6:**
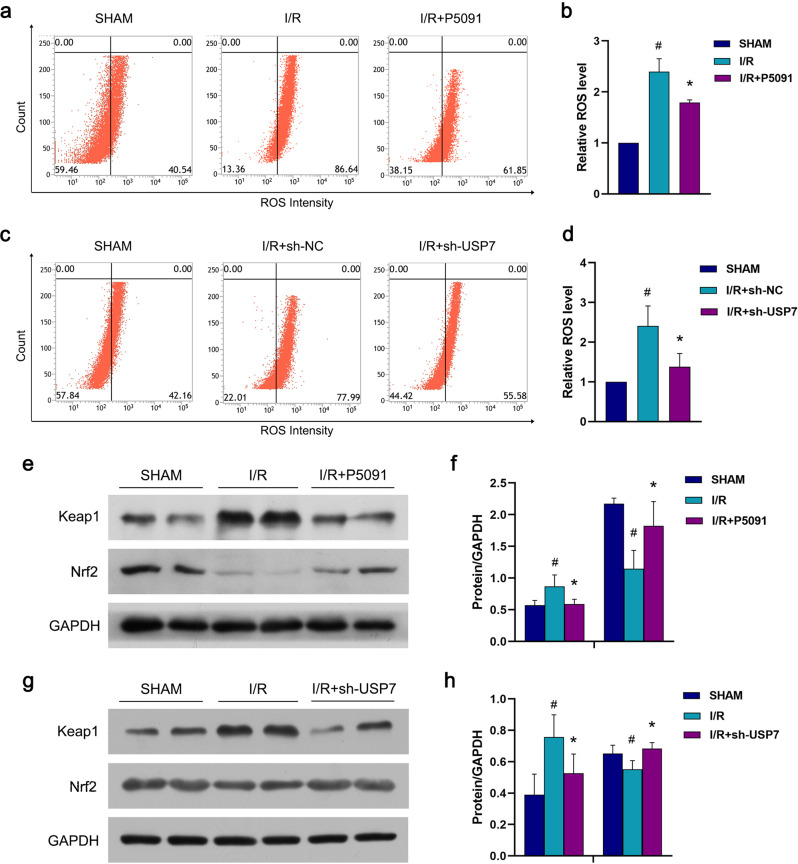
USP7 regulates myocardial apoptosis through Keap1. **a**, **b** P5091 was intraperitoneally injected into mice for three consecutive days before 24 h of ischemia/reperfusion. Flow cytometry was used to measure the fluorescence intensity of ROS. **c**, **d** sh-USP7 was injected into the myocardium of mice, and the myocardial ischemia/reperfusion model was established seven days later. ROS fluorescence intensity was detected by flow cytometry. **e**, **f** The operation and treatment were the same as (**a**, **b**), Immunoblot was used to determine the amounts of Keap1 and Nrf2 proteins. **g**, **h** The operation and treatment were the same as (**c**, **d**), Immunoblot was used to determine the amounts of Keap1 and Nrf2 proteins. ^#^Compared with the SHAM group, *Compared with the I/R group, *P* < 0.05, mean ± standard deviation (*n* = 3).

The pathogenesis of myocardial ischemia/reperfusion injury has been linked to an excessive accumulation of ROS [35]. In this study, ROS generation was significantly increased in the I/R group compared to the SHAM group, whereas ROS levels were decreased following P5091 treatment; similar results were obtained after sh-USP7 treatment (Fig. 6a–d).

Nrf2/Keap1 is an important signaling pathway for reducing myocardial infarct size and maintaining heart function following myocardial ischemia/reperfusion injury. It has been reported that the excessive accumulation of ROS is involved in the pathogenesis of myocardial ischemia/reperfusion injury. To further determine whether inhibition of USP7 activates the Nrf2/Keap1 signaling pathway, we evaluated the changes in Keap1 and Nrf2 expression under the P5091 treatment. Keap1 expression was dramatically elevated in the I/R group; Keap1 interacted with Nrf2 to inactivate it. However, after the P5091 treatment, the tendency was reversed. Keap1 expression was reduced, whereas Nrf2 expression was increased; similar results were obtained after sh-USP7 treatment (Fig. 6e–h). It is supposed that protein degradation causes Keap1 to downregulate and releases Nrf2 to translocate into the nucleus and bind to antioxidant response elements (ARE), activating the antioxidant reaction and reducing ROS production levels.

### USP7 interacts with Keap1 and promotes the degradation of Keap1

**Fig. 7:**
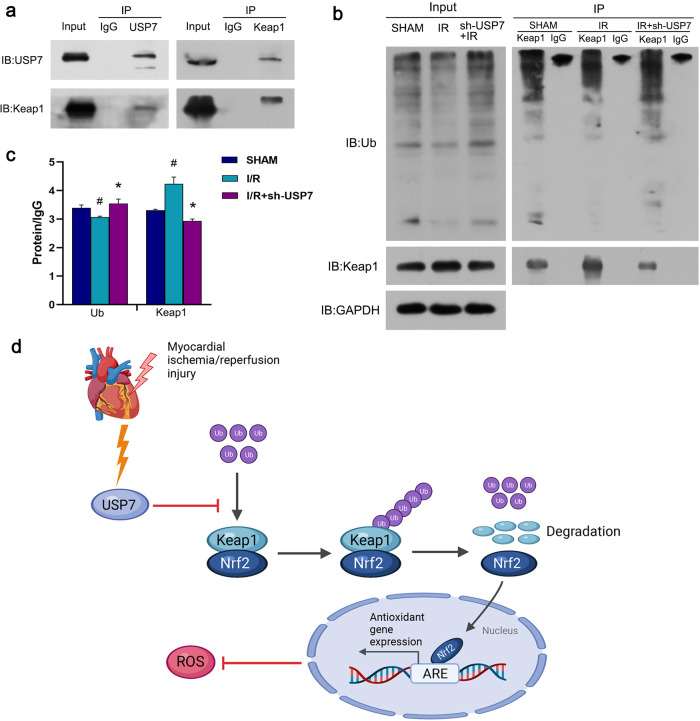
USP7 regulates myocardial ischemia/reperfusion by deubiquitinating and stabilizing Keap1. **a** After 30 minutes of ischemia and 2 h of reperfusion, the mouse heart tissue was taken for Co-IP. USP7’s interaction with Keap1 was investigated using Western blot. **b** Co-IP was performed with Keap1, then immunoblotted with antibodies against ubiquitin (Ub) and Keap1. **b** sh-USP7 was injected into the myocardium of mice, and the myocardial ischemia/reperfusion model was established 7 days later. Immunoblot was used to determine the amounts of ubiquitin (Ub) and Keap1 proteins. **c** Co-IP was performed with Keap1 and IgG, then immunoblotted with antibodies against Ub and Keap1. Quantitative analysis statistics are shown. **d** A proposed mechanism for USP7 to regulate myocardial ischemia/reperfusion via stabilizing the Keap1 level. (Created with BioRender.com).

We predicted that UPS-mediated protein degradation causes Keap1 downregulation at the protein level. We performed co-immunoprecipitation (Co-IP) to investigate whether there is a relationship between Keap1 and USP7. Western blotting showed that Keap1 interacts with USP7 (Fig. 7a). To establish that USP7 regulates Keap1 degradation via deubiquitination, we employed co-IP to quantitatively analyze ubiquitinated Keap1. As expected, the I/R group had considerably less ubiquitin-protein, suggesting that ischemia-reperfusion blocked the ubiquitination pathway of Keap1. On the other hand, USP7 inhibition significantly increased the amount of ubiquitinated Keap1, suggesting that USP7 is a DUB for Keap1, capable of reversing Keap1 ubiquitination and hence boosting Keap1 protein breakdown (Fig. 7b, c).

## Discussion

Our data suggested that USP7 is a negative regulator of myocardial ischemia/reperfusion injury. USP7 acts on Keap1 to reduce ROS production (Fig. 7d). Therefore, targeting the USP7-mediated Keap1 degradation pathway may be a new approach to prevent myocardial ischemia/reperfusion injury.

Myocardial ischemia/reperfusion injury is most commonly seen in patients suffering from acute myocardial infarction (AMI), the leading cause of mortality globally. Myocardial reperfusion with prompt thrombolytic therapy or primary percutaneous coronary intervention (PPCI) is currently considered the most effective therapeutic method for acute AMI. Myocardial reperfusion can produce myocardial injury, diminishing the positive effects of reperfusion, a condition called myocardial ischemia/reperfusion injury. I/R-induced microvascular obstruction and lethal myocardial reperfusion injury are irreversible processes [2, 19].

The complicated pathophysiological process of restoring blood flow after myocardial ischemia, which leads to metabolic dysfunction and structural damage, is known as myocardial ischemia/reperfusion injury. Increasing data suggest that oxidative stress is the primary pathological process causing myocardial injury during reperfusion [36]. During the first few minutes of myocardial reperfusion, various sources produce oxidative stress, and ROS production increases dramatically [37]. Reoxidation of ischemic myocardium results in a certain degree of myocardial injury, which is much more severe than that caused by ischemia alone [8]. Meanwhile, oxidative stress causes cardiac damage and cardiomyocyte death through several pathways, one of which is the Nrf2/Keap1 signaling pathway. Keap1 is both Nrf2’s ally and its opponent. Keap1 regulates Nrf2 ubiquitination and translocation into the nucleus [38]. In addition, the elevation of Nrf2 protein is generally stabilized by Keap1 oxidation or alkylation [39]. Therefore, Keap1 has an indispensable role and can be regarded as one of the important targets for regulating oxidative stress.

The great mass of proteins in mammalian cells is degraded by the UPS, which is hence considered the major pathway of intracellular protein degradation. One of the important functions of UPS is to protect cells from misfolded or damaged proteins [40, 41]. In this study, abnormal elevation of Keap1 protein during myocardial ischemia/reperfusion was related to UPS disorder, which was confirmed by the return of Keap1 protein to normal level after treatment with USP7 inhibitor P5091. Studies have linked aberrant USP7 expression and activity to various disorders, including apoptosis, inflammation, and life cycle regulation [30–32]. Furthermore, USP7 significantly increases under hypoxia in cardiomyocytes, while its inhibition reduces the myocardial damage caused by continuous hypoxia, which indicates that USP7 is effective in myocardial damage [33]. In this study, we found the relationship between USP7 and Keap1, and demonstrated that USP7 interacts with Keap1 to reduce abnormally elevated Keap1 protein to a stable state through the proteasome degradation pathway, which may be related to the release of Nrf2 into the nucleus to trigger antioxidant responses, thus reducing ROS production and alleviating myocardial ischemia/reperfusion injury. As a result, USP7 may become a novel therapeutic target for myocardial ischemia/reperfusion injury.

## Materials and methods

### Animal experimental protocol

All animal procedures were approved by the Animal Ethics Committee of Guangzhou Medical University’s Second Affiliated Hospital, and studies were carried out in accordance with the National Institutes of Health’s “Guidelines for the Care and Use of Laboratory Animals” (Eighth Edition).

C57BL/6 mice (6–8 weeks old, male) were provided by the Guangdong Experimental Animal Center. All the animals were housed in an environment with a temperature of 22 ± 1 °C, a relative humidity of 65 ± 5%, and a light/dark cycle of 12/12 h and given food and water ad libitum. All animals were anesthetized with isoflurane (1.5–2%) and then euthanized by cervical dislocation once the experiment was completed.

To establish a myocardial I/R model, mice were first anesthetized and,connected to the ventilator by endotracheal intubation, after which chest skin was disinfected with 75% alcohol and iodophor. The skin layer was cut at the 3–4 intercostal gap on the left border of the sternum using ophthalmic scissors, the fascia and muscle were separated layer by layer with ophthalmic forceps, and the ribs were secured with a chest expander. The pericardium was gently opened using ophthalmic forceps to expose the heart. The left atrial appendage and the pulmonary artery cone were then found. Then, a 9‐0 ophthalmic suture was used to ligate the left anterior descending coronary artery (LAD) at 2–3 mm of the lower margin of the left atrial appendage. The visualization of pale color in the myocardium distal to the occlusion was an indicator of effective LAD occlusion. After 45 min of ischemia, blood flow was restored by releasing the ligature. Ligation was not released in the AMI group.The LAD was not ligated in the SHAM group.

Male mice were randomly divided into three groups: SHAM group, I/R group, and I/R + P5091 or I/R + shUSP7 group. No LAD ligation was performed in the SHAM group, while the rest of the operation was indicated for the I/R group. For the treatment group, mice were intraperitoneally injected with USP7 inhibitor P5091 (10 mg/kg) for three consecutive days before I/R operation, while the other groups were intraperitoneally injected with sterile saline. On the other hand, after thoracotomy of mice, 10 µl adenovirus (1 × 10^10^ VP/ ml) was injected at four points in the apex of the heart with a 30 G injection needle, while Scramble shRNA was injected in the other groups. One week later, thoracotomy was performed again for the I/R operation [42].

### Materials

P5091 was produced by Selleck (USA). Cell signaling technologies provided anti-USP7 and anti-bax antibodies (USA). Proteintech (China) produced anti-Keap1. Bioworld (Bioworld Technology, USA) provided anti-Nrf2 and anti-GAPDH antibodies. Antibodies produced against ubiquitin were bought from Life Technologies, as was the Co-IP assay kit (USA). Invitrogen produced the TRIzol reagent (USA). Takara Biomedical Technology (Beijing) Co., Ltd. provided the PrimeScript II 1st Strand cDNA Synthesis Kit. Keygen created the Cell Cycle Detection Kit (China). Santa Cruz Biotechnology provided the enhanced chemiluminescence (ECL) kit (USA). ROS Assay Kit was bought from Beyotime Biotechnology (China). Cusabio Technology (China) provided ELISA Kit. TUNEL Assay Kit was acquired from Roche (China). For immunoblot, immunofluorescence, and Co-IP assays, antibodies were diluted at 1:1000, 1:2500, 1:500, and 1:50, respectively.

### Echocardiography

The limbs of the mouse were fixed, after which 2% isoflurane was used to anesthetize the mouse. Anesthesia continued until the end of the Echocardiography. It should be noted that this process did not affect the heart rate of the mice. Echocardiography was performed using an M-mode echocardiogram Vevo 2100 (VisualSonics, Toronto, Canada). A 250 MHZ probe was placed on the left chest of the mouse parallel to the anterior midline of the mouse, with the tip of the probe facing. Consequently, the function and structure of the heart were evaluated to obtain a short-axis view and a clear image. Finally, the left ventricular EF% and FS% were measured.

### Flow cytometry assay

After squeezing the blood, heart tissue was cut into tissue 1 mm pieces that were placed in a DMEM medium. The tissues were then ground, centrifuged for 5 s in a mini centrifuge, and filtered on a 300-mesh filter. This step was repeated until the tissue mass disappeared completely. The DCFH-DA solution was then prepared according to ROS Assay Kit (Beyotime Biotechnology, China), shaken and mixed. The solution was then mixed with obtained cells cultured in a 37 °C incubator for 30 min, turning the dish every 5 min. Next, samples were centrifuged for 5 min at 1000 rpm, and the supernatant was dscrarded. Samples were washed in PBS and then centrifuged again to discard the supernatant. Finally, samples were mixed with 200 µl of PBS and analyzed by FITC at 488 nm/525 nm (BD FACSVerse, USA).

### TUNEL staining

The heart was rapidly removed and fixed in paraformaldehyde for 24 h, after which 5μm paraffin slices were produced, and proteinase K working solution was poured into the circle to cover the tissue and incubated for 22 min at 37 °C. The rupture fluid should then be dropped over the tissue, incubated at room temperature for 20 min, and then washed. After the slices had been gently dried, the buffer was poured dropwise to cover the tissue and incubated for 10 min at room temperature. Samples were then incubated at 37 °C for 2 h after adding the required amount of TDT enzyme, dUTP, and mixing the buffer at a ratio of 1:5:50. The DAPI staining solution was then applied dropwise and incubated in the dark for 10 min at room temperature. Samples were then mounted with an anti-fluorescence quenching mounting plate. The slices were examined under a fluorescent microscope, and photographs were taken. Under UV excitation, the positive cells appeared green.

### ELISA assay

All reagents were placed at room temperature for 30 min. Standard product holes and sample holes were then separately set. A 100 μl of standard or mouse serum sample was added to each well and incubated for 2 h at 37 °C. Consequently, the liquid was discarded and replaced with 100 μl of biotin-labeled antibody working solution; samples were then incubated for 1 h at 37 °C. Then, the liquid was discarded, and each well was refilled with 100 μl of horseradish peroxidase-labeled avidin working solution for 1 h at 37 °C. After the liquid was discarded, a 90 μl of substrate solution was added to each well, and samples were placed in the dark at 37 °C for 15–30 minutes. To halt the reaction, a 50 μl of stop solution was added to each well. Within 5 min after the reaction’s completion, a microplate reader was used to determine the optical density value of each well at 450 nm wavelength.

### Western blot and Co-IP analysis

The blood of the heart tissue was washed with PBS, and tissue blocks of appropriate size were cut out with scissors and put into EP tubes. A 4 mm large steel balls and 3 mm small steel balls were added to each tube, and an appropriate amount of tissue lysate was added for cracking. Samples were then homogenized in a high-speed tissue grinder (Servicebio KZ-II, China), and the supernatant was obtained by centrifugation as tissue protein. SDS-polyacrylamide gels with various concentrations of sodium dodecyl sulfate (SDS) were electrophoretically resolved, and PVDF membranes were electro-transferred. Different molecular weights of proteins were electrophoretically resolved with sodium dodecyl sulfate (SDS)-polyacrylamide gels, and polyvinylidene difluoride (PVDF) membranes were electro-transferred. Membranes were sealed with 5% skimmed milk for 1 h and then washed three times with PBST, each time for five minutes. Then, membranes were incubated with primary antibody overnight at 37 °C and then secondary antibody at room temperature for 1 h. Finally, samples were detected by the ECL color method.

For protein interaction (co-IP) experiments, the antibody and dynabeads were first mixed for 16–22 h. Then, the tissue lysate was added for 1 h. PBST was rinsed three times; then, the dynabeads were removed, after which western blotting was performed.

### EVANS BLUT-TTC double staining experiment

The anterior descending coronary artery was ligated at the primary surgical site after 24 h of I/R, and 2% Evans blue staining solution was injected through the jugular vein with an insulin needle. After the mouse’s limbs, lips, and skin turned blue, the heart was gently cut using forceps. The heart was then cut it into 5–6 pieces at room temperature, with a thickness of about 2 mm, which were incubated in 1% TTC staining solution for 15–30 min at 37 °C in the dark. The distinction was performed according to the color of the surface of the heart tissue (white is the infarct area, red is the ischemic area, and blue is the non-ischemic and non-infarct area). After images were collcted, Image-pro Plus software was used to calculate the proportion of each area.

### Immunohistochemical (IHC) staining

The heart was embedded in paraffin and sliced after being preserved with 4% paraformaldehyde. In simple terms, the slices were dewaxed and hydrated for antigen repair. To inactivate endogenous peroxidase, 3% hydrogen peroxide was applied and incubated at room temperature for 20 min. Then 10% goat serum was added to block non-specific antigen binding for 30 min. The primary antibody (1:1000) was incubated overnight at 4 °C, followed by a 30 min incubation at room temperature with the secondary antibody (1:2000). Then, it was stained with diaminobenzidine and observed under a microscope. Finally, it was rinsed with tap water, stained with hematoxylin, rinsed again, and then dry and mounted under glass coverslips.

### PCR analysis

After I/R, ventricular heart tissues were collected, and RNAs were extracted from them using a TRIzol reagent according to the manufacturer’s instructions, as we previously reported [43]. The first-strand cDNA was synthesized from an equivalent number of RNAs using the PrimeScript II 1st Strand cDNA Synthesis Kit. PCR primers are as follows: USP7: F: 5ʹ-TCAAGTCTCAAGGTTATAGGG-3ʹ; R: 5ʹ-CTGTTCTCAAAGTCCGTG-3ʹ; GAPDH: F: 5ʹ-ACCCAGAAGACTGTGGATGG-3ʹ; R: 5ʹ-ACACATTGGGGGTAGGAACA-3ʹ. Real-time quantitative PCR was used to detect the mRNA levels of the targeted gene.

### Masson’s trichrome staining

This experiment was performed following a previously decribed approach [44]. Tissues were fixed with Bouin’s or Zenker’s liquor, and stained with Harris hematoxylin, acid ponceau, and aniline blue. Microscopically, myocardial fibers were red, and collagen fibers were blue. The area proportion of collagen fiber was quantitatively analyzed by ImageJ software.

### Statistical analysis

Data are shown as mean ± SD from three independent experiments. Using unpaired Student’s *t*-test or one-way ANOVA was applied where appropriate to evaluate statistical probabilities. Statistical analysis was performed using GraphPad Prism8.0 software and SPSS 16.0. A *P* value of <0.05 was considered to be statistically significant.

## Supplementary information

original western blots

## Data Availability

All the data and material supporting the conclusions were included in the main paper.
