# Supplementary material for: Ubiquitin-specific protease 7 regulates myocardial ischemia/reperfusion injury by stabilizing Keap1
Source: Cell Death Discov. 2022 Jun 16;8:291. doi: 10.1038/s41420-022-01086-2 (PMC9203583; doi:10.1038/s41420-022-01086-2)
Supplement: Supplementary file 1 — original western blots [file 41420_2022_1086_MOESM1_ESM.pdf]

The original western blots were listed as following.

Figure1 a

USP7

GAPDH

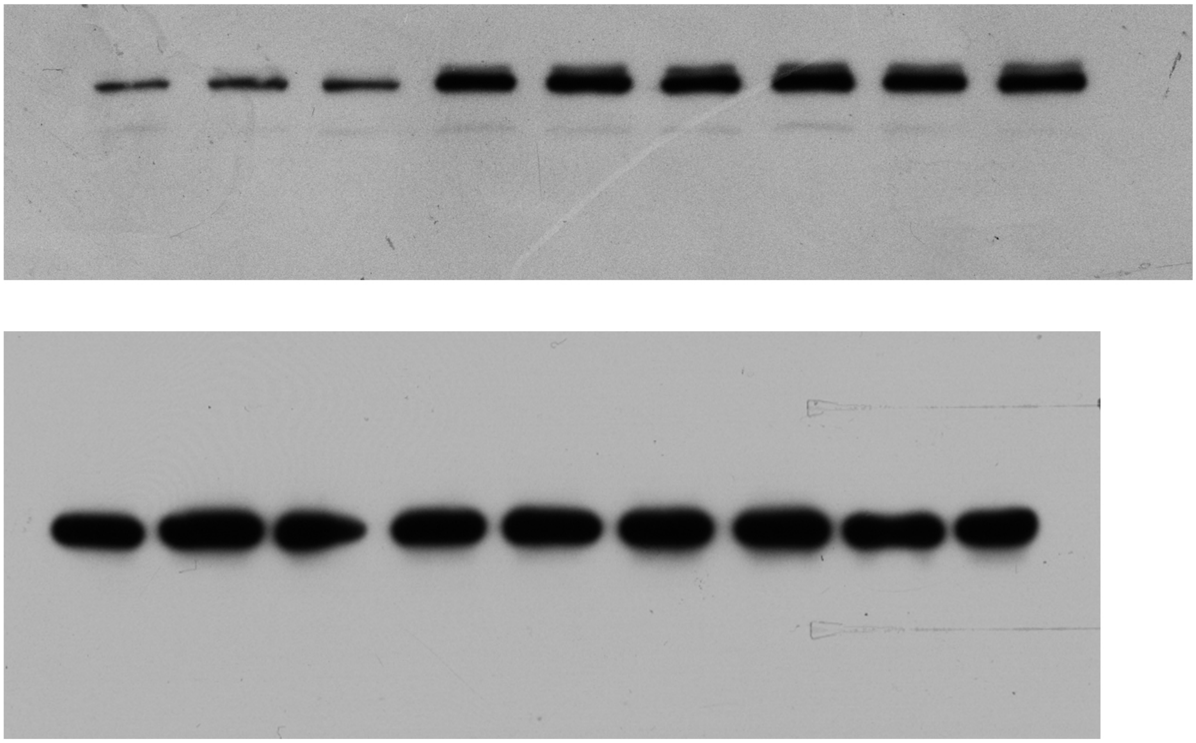

Figure1 c

USP7

GAPDH

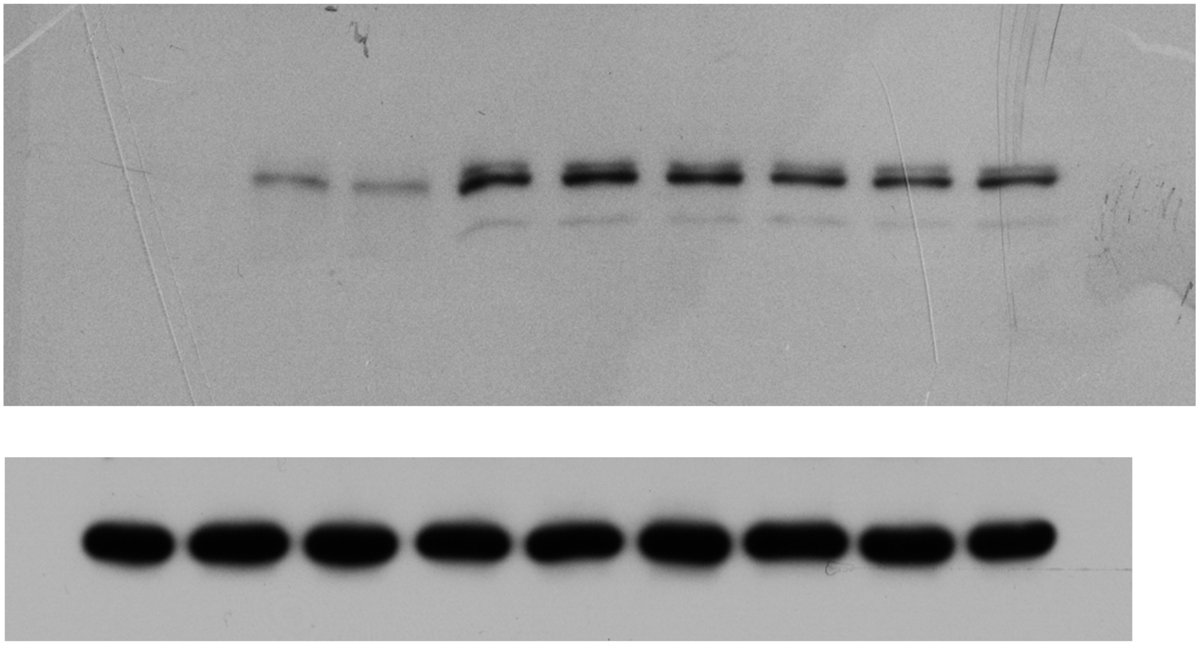

Figure2 a

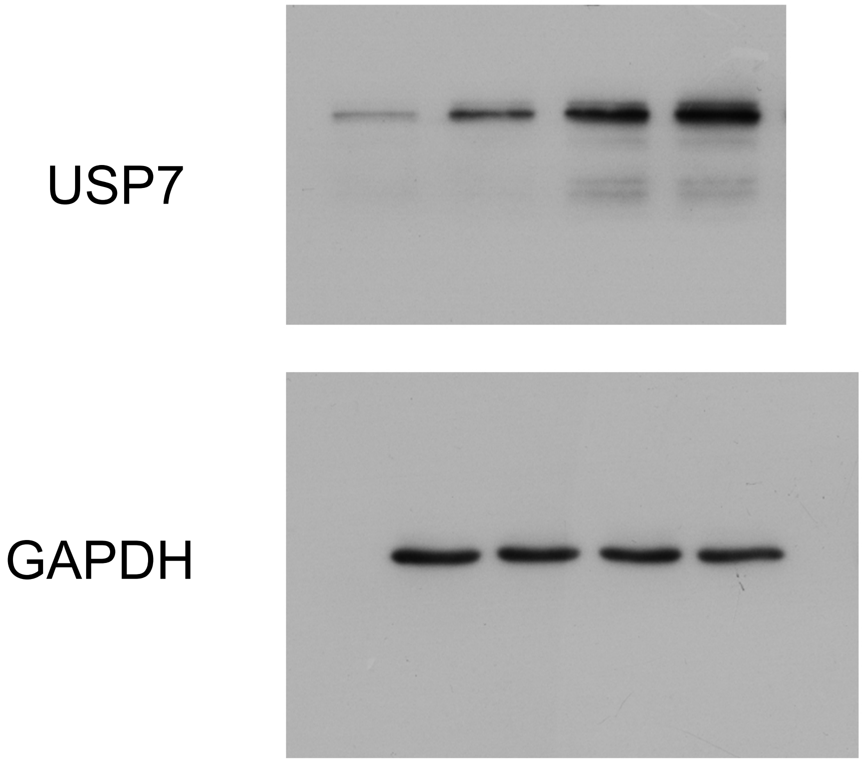

Figure3 a

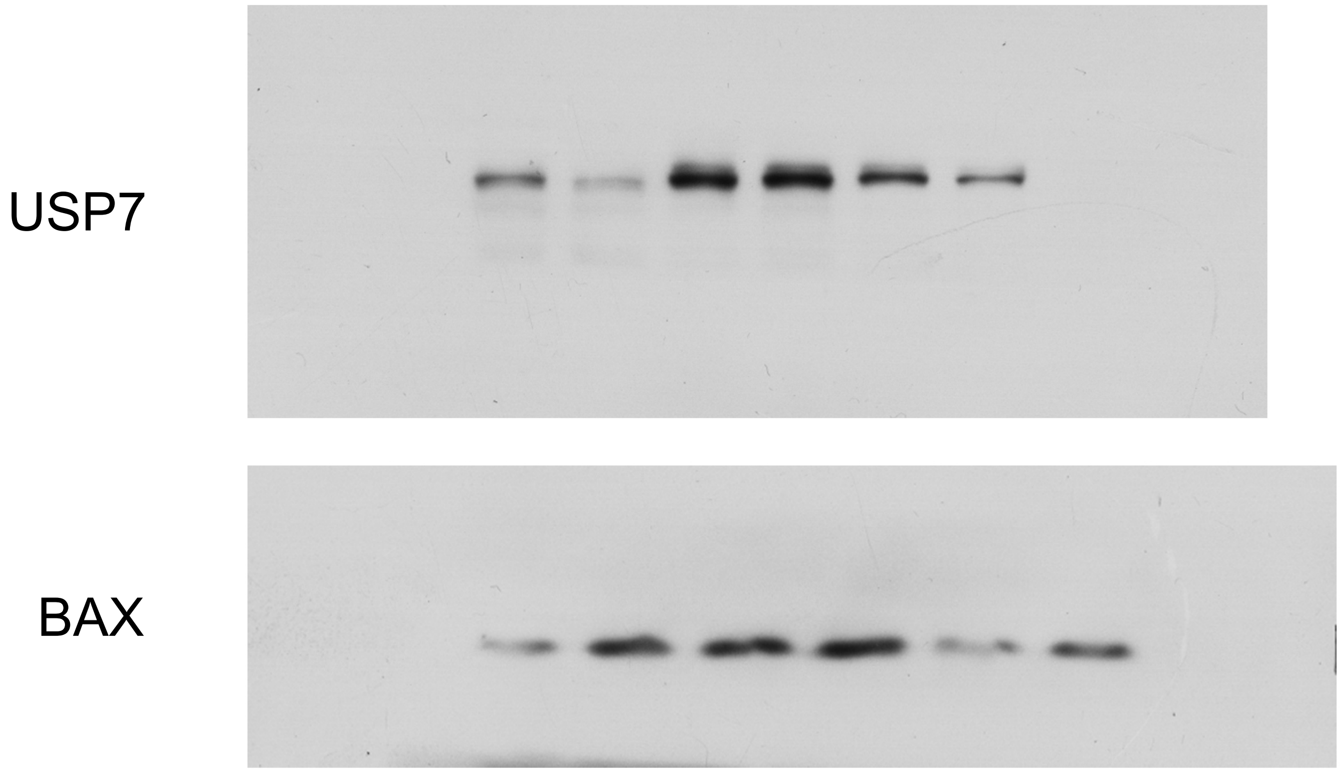

GAPDH

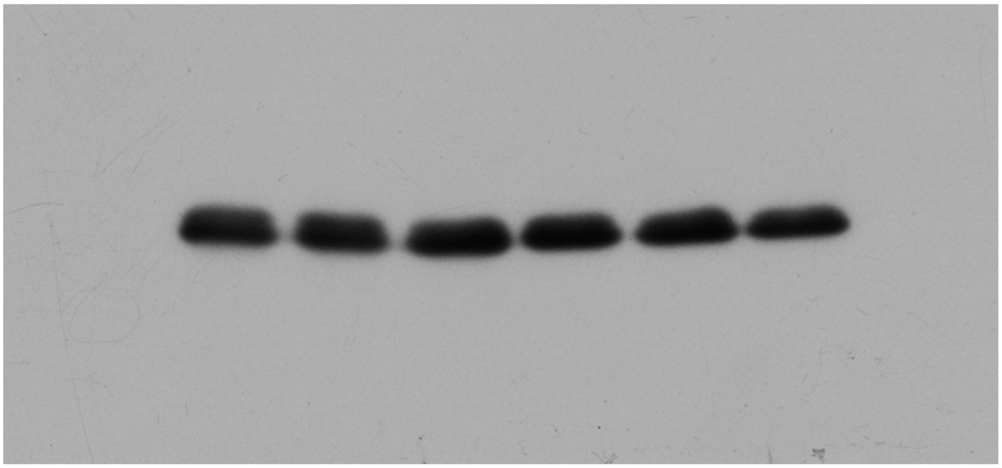

Figure3 c

USP7

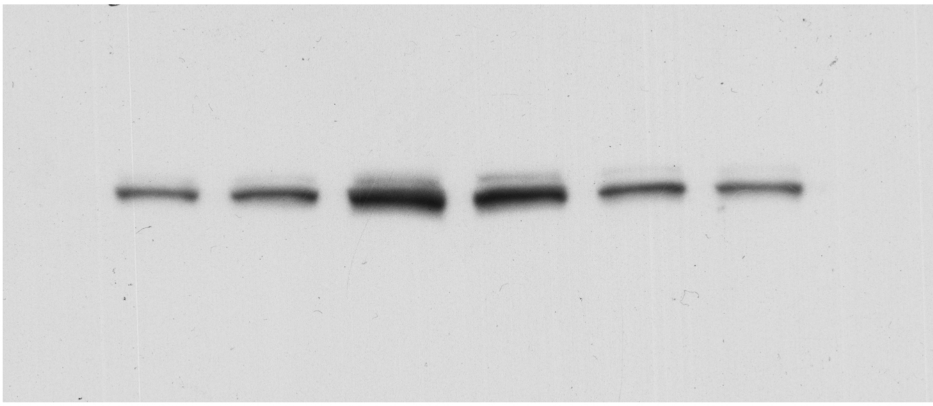

BAX

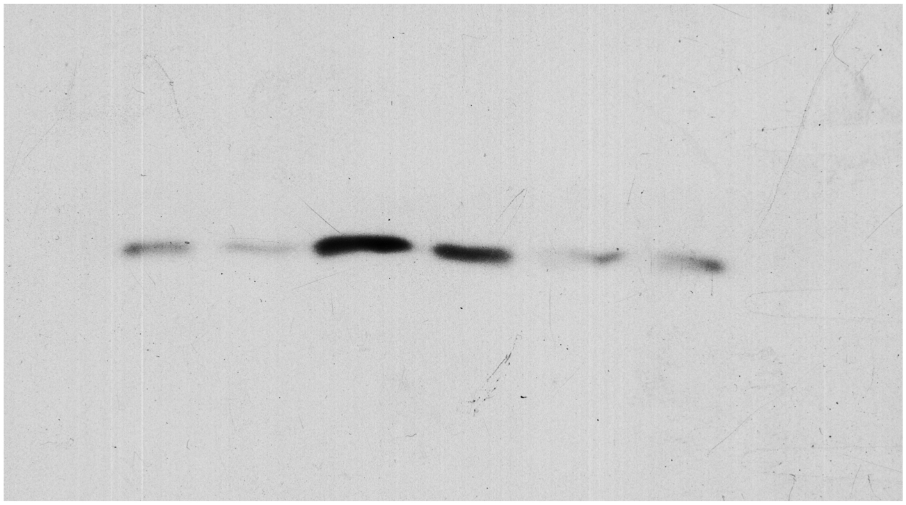

GAPDH

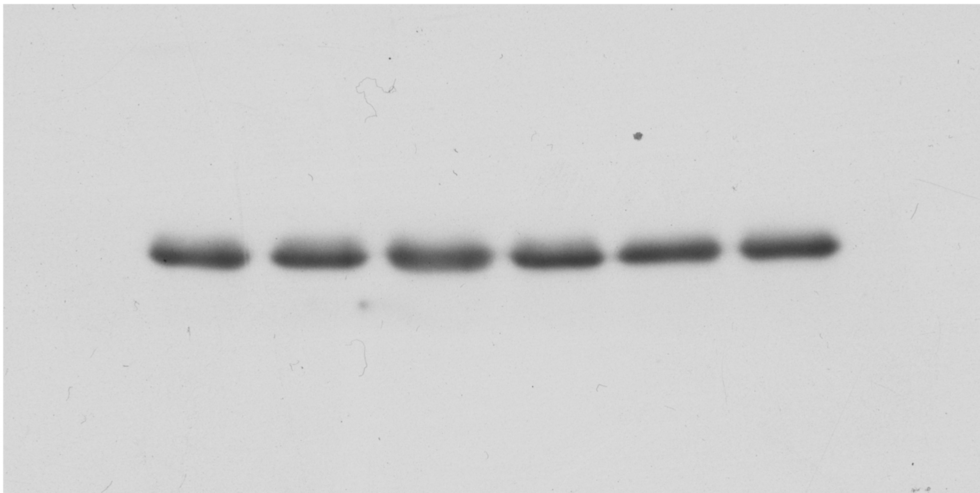

Figure6 e

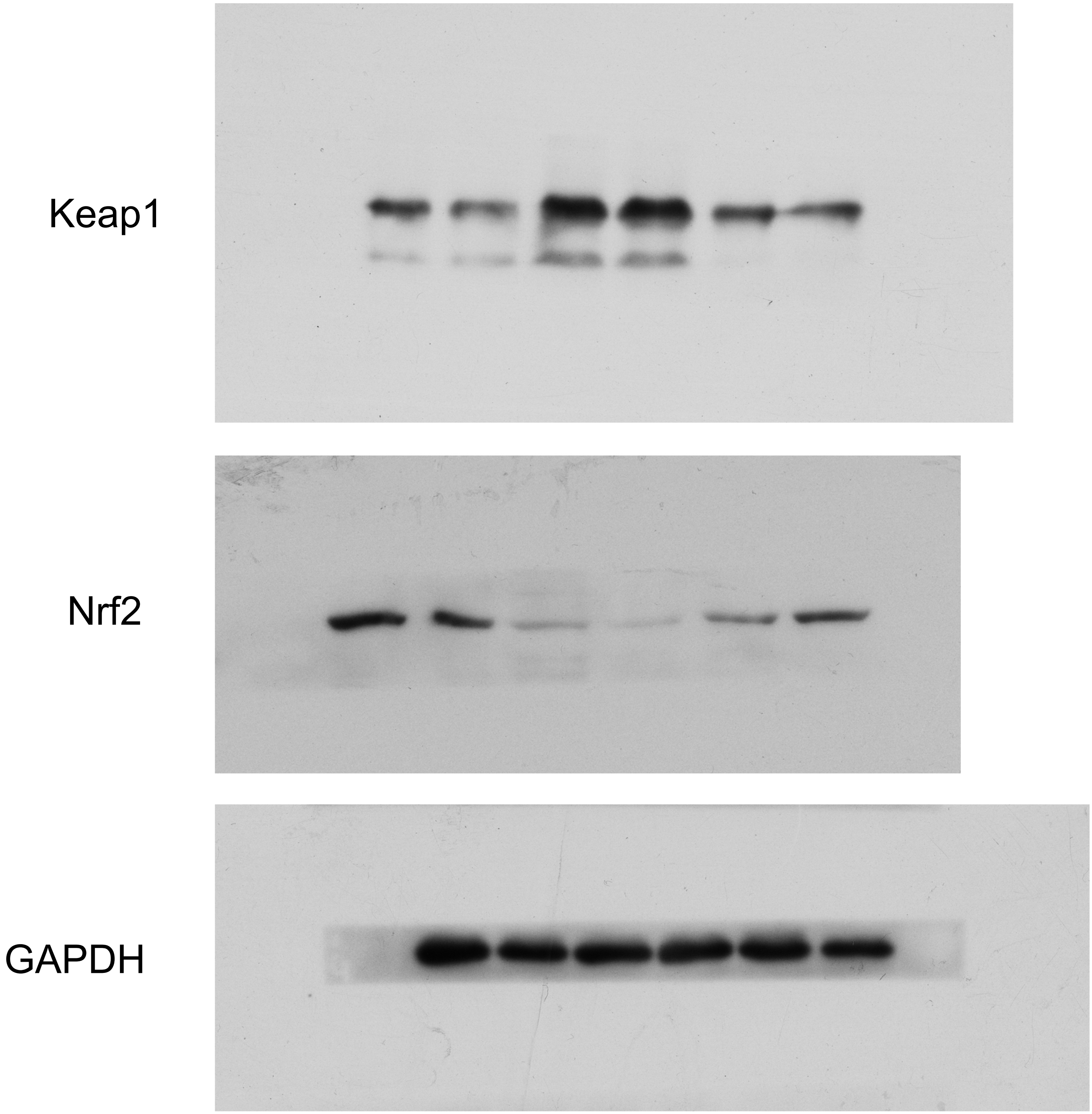

Figure6 g

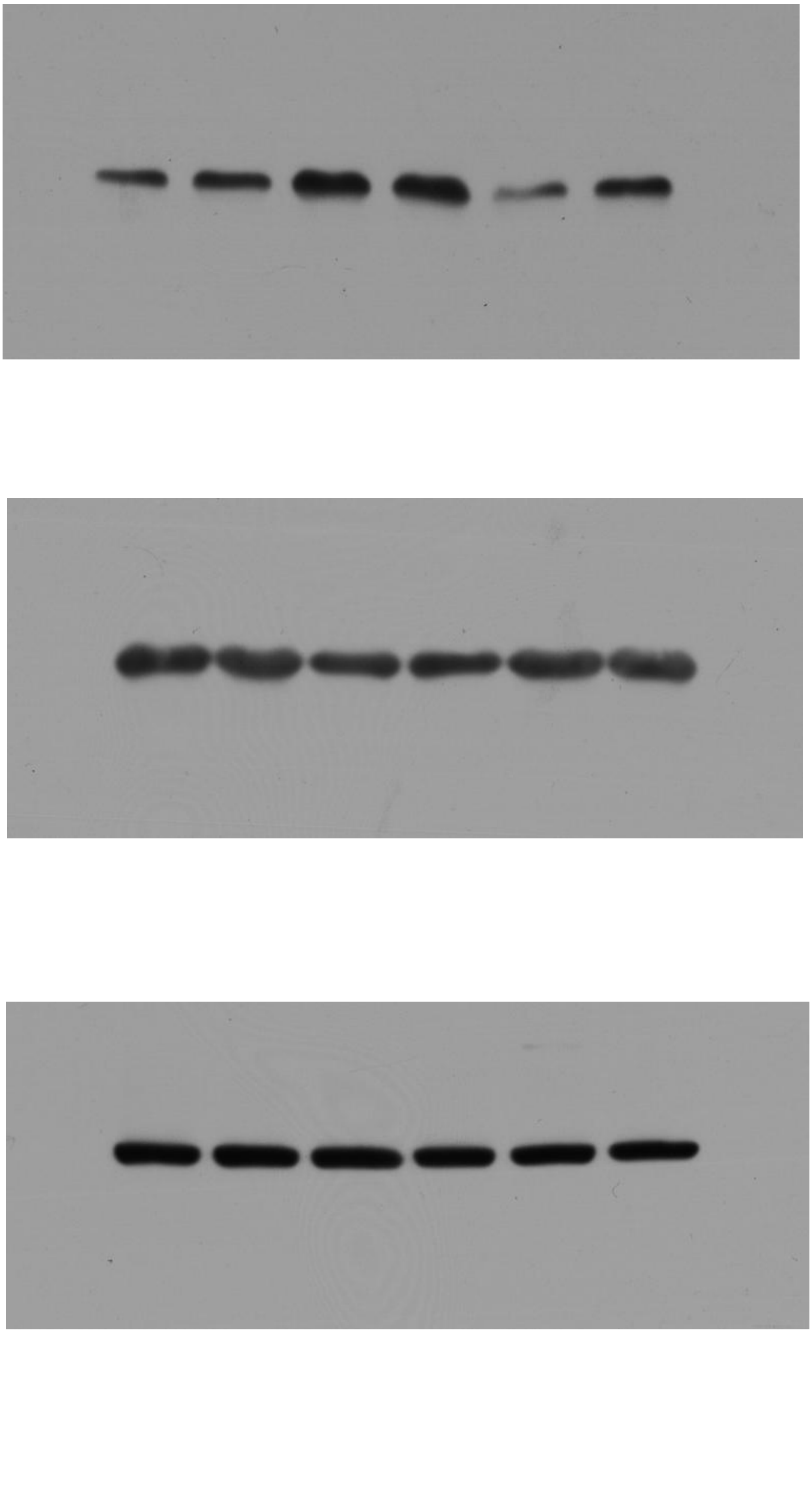

Keap1

Nrf2

GAPDH

Figure7 a

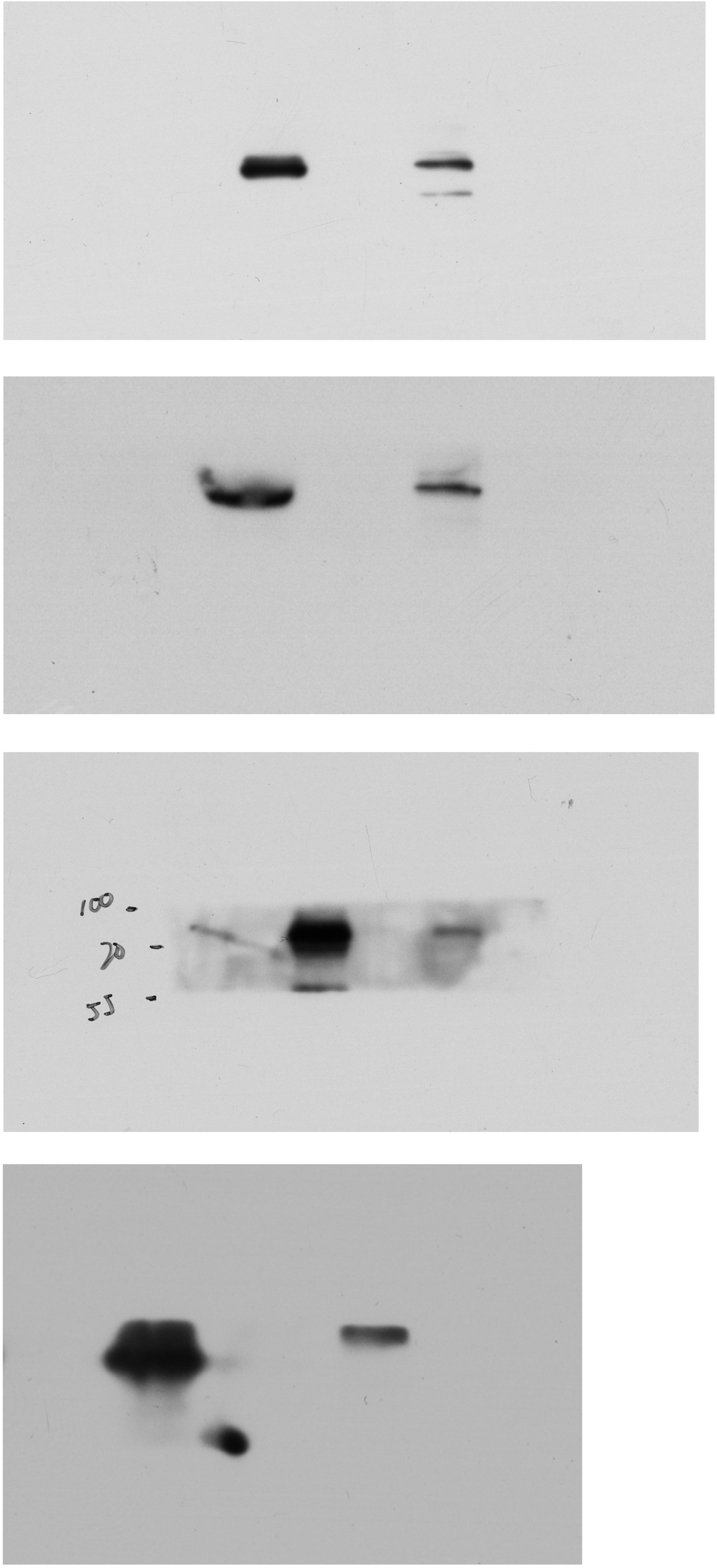

USP7

USP7

Keap1

Keap1

Figure7 b

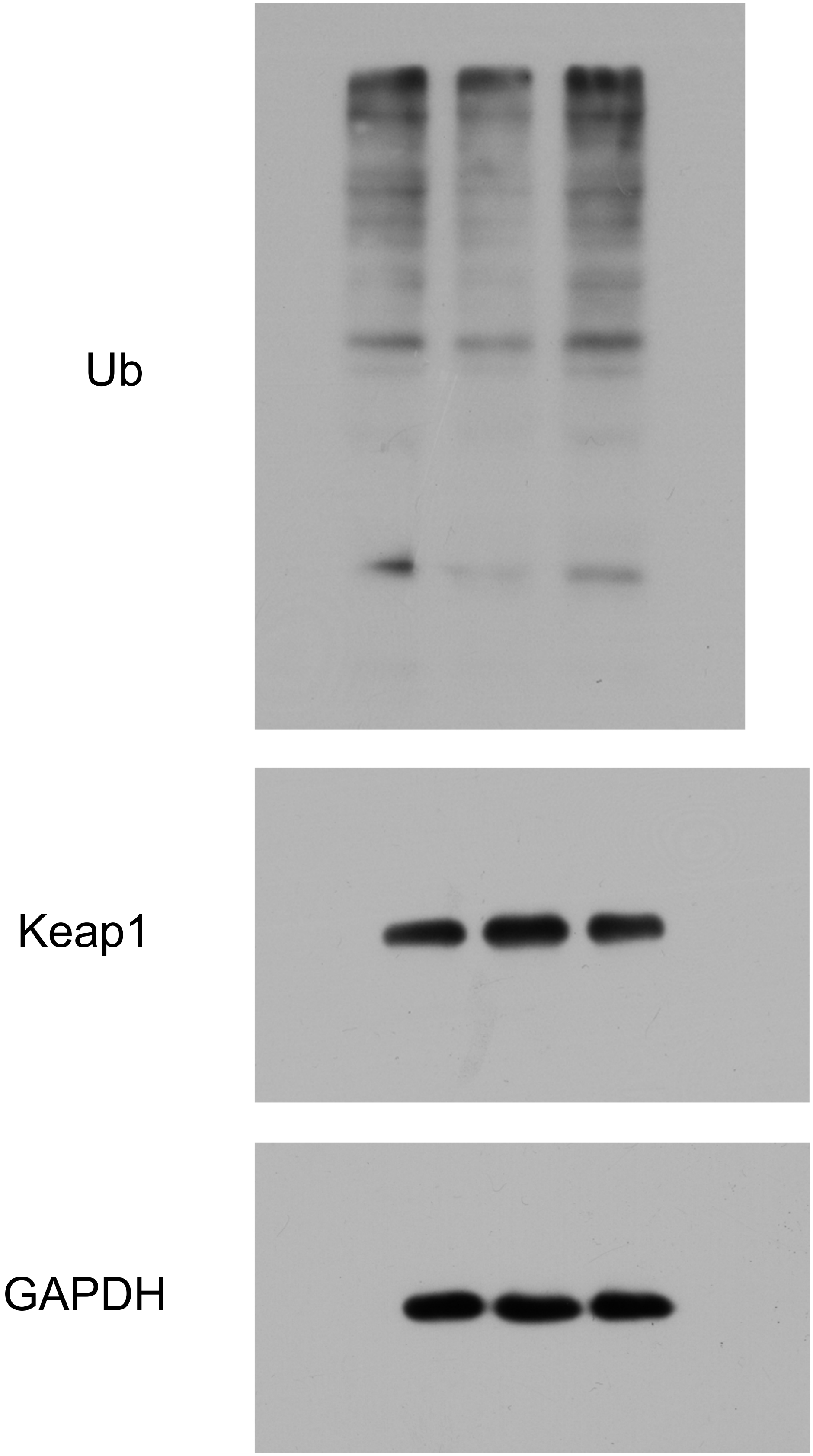

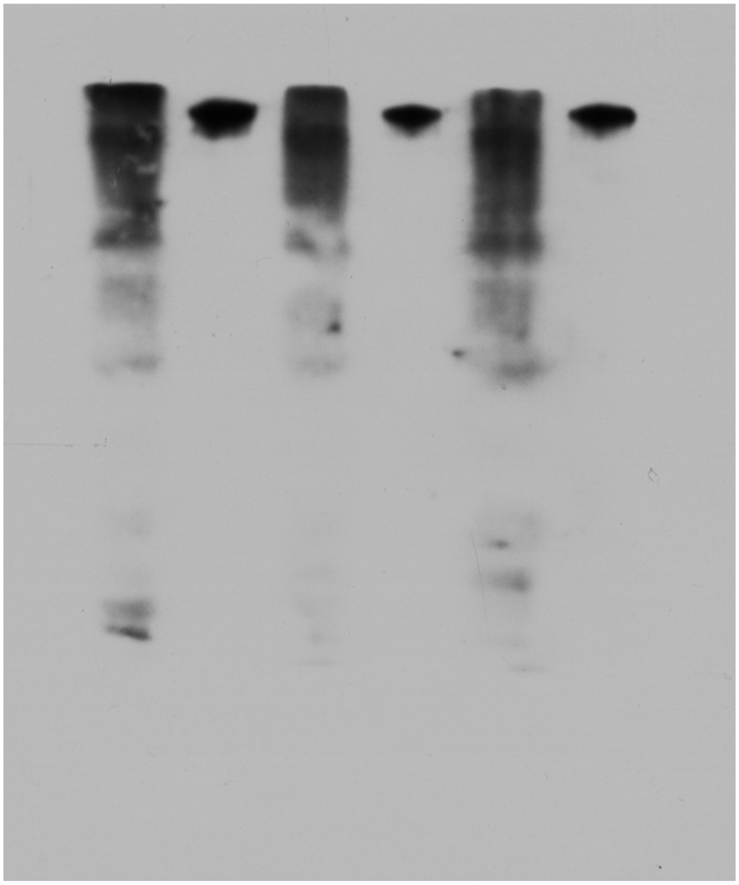

Ub

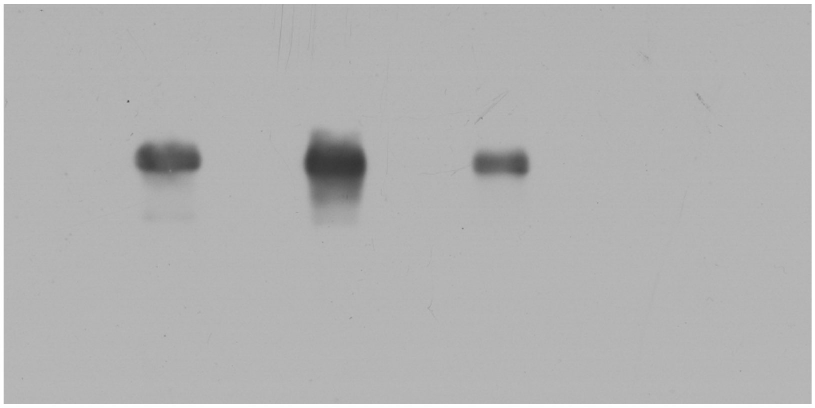

Keap1
